# Prognostic value of cardiac magnetic resonance–derived global longitudinal strain in LGE-negative dilated cardiomyopathy

**DOI:** 10.1371/journal.pone.0345077

**Published:** 2026-03-27

**Authors:** Tian-Yue Zhang, Tian Lan, Ling-Li Wang, Yu-Cong Zheng, Xin-Yi Feng, Fei-Yao Wang, Fan Zhang, Hua-Yan Xu, Xi Liu, Zhi Yang, Rui Li

**Affiliations:** 1 Department of Radiology, Affiliated Hospital of North Sichuan Medical College, Nanchong, China; 2 Department of Radiology, Chengdu Fifth People's Hospital, Chengdu, China; 3 Department of Magnetic Resonance Imaging, Fuwai Hospital, National Center for Cardiovascular Diseases, Chinese Academy of Medical Sciences and Peking Union, Beijing, China; 4 Department of Radiology, Tsinghua University Hospital, Tsinghua University, Beijing, China; 5 Department of Radiology, West China Second University Hospital, Sichuan University, Chengdu, China; 6 Department of Radiology, Peking University Cancer Hospital, Institute Key Laboratory of Carcinogenesis and Translational Research (Ministry of Education), Beijing, China; Scuola Superiore Sant'Anna, ITALY

## Abstract

**Background:**

Dilated cardiomyopathy (DCM) is a major cause of heart failure and sudden cardiac death (SCD), with a 5-year survival rate of approximately 45%–50%. Current risk stratification is predominantly dependent on left ventricular ejection fraction (LVEF), which has limited sensitivity and specificity. Hence, more effective biomarkers should be used in late gadolinium enhancement (LGE)-negative patients.

**Methods:**

A total of 378 consecutive patients with LGE-negative DCM were enrolled from four hospitals between December 2016 and December 2022. Cardiac magnetic resonance imaging-derived strain parameters (global radial strain, global circumferential strain, and global longitudinal strain [GLS]) were assessed against the primary (SCD and related events) and secondary (heart failure, appropriate implantable cardioverter-defibrillator therapy) endpoints. Internal validation was performed using stratified bootstrap resampling with Harrell’s optimism correction to report the optimism-corrected C-index. Data were accessed for research purposes from 15/06/2023–30/12/2023, and all records were de-identified prior to analysis.

**Results:**

Over a median follow-up of 59.78 months, 35 (9.26%) and 72 (19.0%) patients presented with the primary and secondary endpoints, respectively. Based on the multivariate Cox analysis, GLS, LVEF, and age were independent prognostic factors. However, only GLS (HR = 1.37; *P* = 0.041) remained significant in the LVEF <20% subgroup. A model integrating GLS and LVEF had a better discrimination ability for SCD than LVEF strata alone (apparent C-index 0.756 vs 0.714, *P* < 0.001). This advantage persisted after bootstrap internal validation (B = 1000; optimism-corrected C-index 0.754 vs 0.711, Holm adjusted *P* = 0.048). Further inclusion of age and New York Heart Association (NYHA) classification enhanced the model’s performance (model 4): apparent C-index 0.801; optimism-corrected C-index 0.785).

**Conclusion:**

GLS is an independent predictor of SCD-related events in LGE-negative DCM. Incorporating GLS with conventional indicators such as age, NYHA classification, and LVEF significantly enhances prognostic discrimination and model robustness, indicating potential value for future clinical risk stratification.

## 1. Introduction

Dilated cardiomyopathy (DCM), which is characterized by left ventricular dysfunction, affects approximately 0.04% of adults, and it is the most common type of cardiomyopathy [1]. However, the prognostic outcomes of DCM vary widely among individuals, with 5-year mortality rates of 20%–50% [2,3], thereby underscoring the urgent need for reliable risk stratification markers [3]. Replacement myocardial fibrosis, as detected on late gadolinium enhancement (LGE) on cardiac magnetic resonance imaging (CMR), is a major contributor to adverse cardiovascular events, particularly ventricular arrhythmias (VAs) and sudden cardiac death (SCD) [4–7]. Nevertheless, LGE is only detectable in approximately 40% of patients with DCM [8,9], highlighting the need for alternative prognostic indicators in a substantial proportion of LGE-negative individuals who remain at substantial risk and require precise risk assessment. Currently, left ventricular ejection fraction (LVEF) remains a key parameter for risk stratification in patients with DCM [10–12], reflecting global cardiac function and contractile performance. However, LVEF has significant limitations, particularly its reduced predictive power for adverse events in patients with preserved ejection fraction, which requires the development of more comprehensive assessment approaches. Importantly, a significant reduction in LVEF has not been observed in the majority of patients with DCM who experienced SCD events over the last 15 years, indicating that reliance on LVEF alone does not yield highly precise prognostic information [13,14]. In a multicenter cohort study, Di Marco et al. used a multiparametric stratification model integrating both LVEF strata (≤20%, 21%–35%, > 35%) and LGE status. Results showed that even among patients with severely reduced LVEF (≤20%), those who are LGE negative still exhibited a significant residual risk (annual event rate: 2.7% vs. 8.1% in LGE-positive counterparts). Based on this study, two important needs were identified: improved risk discrimination among patients with preserved LVEF via myocardial tissue characterization and more refined substratification in populations with severely reduced LVEF using advanced phenotypic profiling [14].

In recent years, cardiac magnetic resonance feature tracking (CMR-FT) has facilitated the assessment of myocardial deformation using conventional cine cardiac sequences. Myocardial strain parameters derived from CMR-FT can detect subtle myocardial injuries even before the appearance of LGE [15–17]. Hence, CMR-FT can be a valuable tool for the early evaluation of myocardial dysfunction—an independent risk factor for adverse events in patients with DCM [18,19]. However, with increasing research advancements, it has become clear that relying on a single parameter for prognostic assessment in DCM is insufficient, as it fails to comprehensively identify patient outcomes. In particular, a few studies have addressed the prognosis of LGE-negative patients, a subgroup that requires special attention due to the absence of conventional fibrosis markers typically used in risk stratification. To address this unmet need, the current study aimed to explore the prognostic value of myocardial strain parameters in patients with LGE-negative DCM. Further, novel risk stratification models were developed by integrating myocardial strain measurements with different LVEF strata, thereby offering a more refined approach to prognostic evaluation in this clinically important population.

## 2. Materials and Methods

### 2.1. Study population

**Fig 1:**
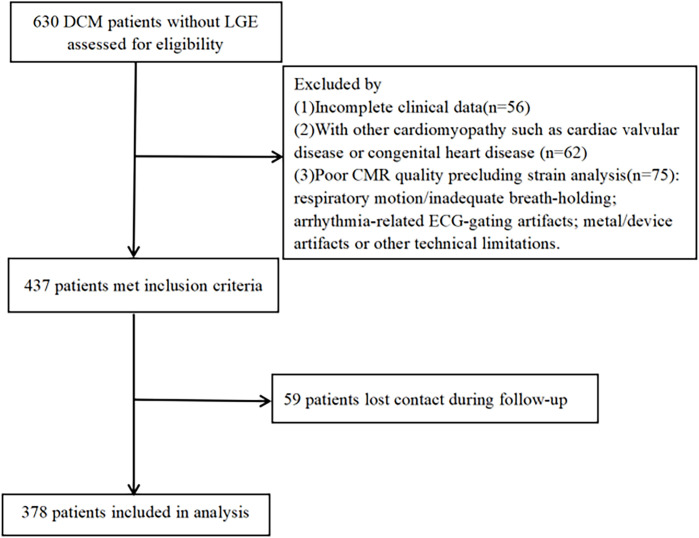
Flowchart shows patient inclusion. DCM = dilated cardiomyopathy, LGE = late gadolinium enhancement, CMR = cardiac magnetic resonance.

Newly diagnosed DCM inpatients were consecutively enrolled from four hospitals between December 2016 and December 2022 (Fig 1). All participants underwent baseline CMR during the index hospitalization as part of the initial diagnostic work-up. The local ethics committee approved the study protocol (approval no. 2021ER134−1) and waived the need for informed consent due to the study’s retrospective design. Data were accessed for research purposes from 15/06/2023–30/12/2023, and all records were de-identified prior to analysis. DCM was diagnosed according to the 2023 ESC guidelines [1], requiring an LVEF <45%, LV dilation (LV end-diastolic volume index >117% of predicted), and exclusion of other causes (e.g., coronary artery disease with ≥50% stenosis, primary valvular heart disease, hypertension, and tachyarrhythmia). The exclusion criteria included ischemic cardiomyopathy, acute/subacute myocarditis, other types of cardiomyopathies, and MRI contraindications. Patients were also excluded if CMR image quality was insufficient for reliable feature-tracking/strain quantification (e.g., severe respiratory motion, arrhythmia-related ECG-gating artifacts, or device/metal-related artifacts, including cases with low signal-to-noise or incomplete LV coverage) (Fig 1).

### 2.2. CMR protocols and image analysis

**Fig 2:**
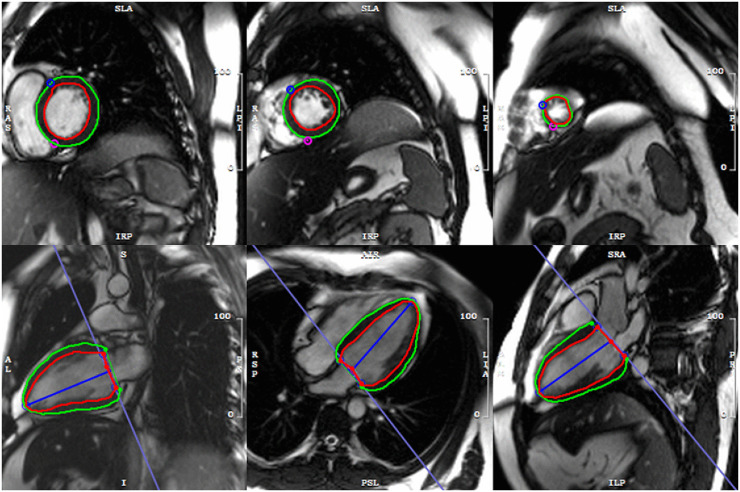
A schematic diagram illustrating the measurement of myocardial strain at the end of left ventricular diastole.

In all cases, CMR imaging was performed using 3.0-T scanners equipped with vector electrocardiography and respiratory gating systems. A balanced steady-state free precession sequence was used to acquire cine images in the short-axis, two-chamber, three-chamber, and four-chamber views of the left ventricle. LGE imaging was conducted 10–15 min after the intravenous administration of gadolinium-based contrast agents, utilizing a phase-sensitive inversion recovery sequence to obtain short-axis and four-chamber images. Images were analyzed offline using a dedicated software (CVI 42, Circle Cardiovascular Imaging, Inc., Calgary, Canada) by two blinded, experienced radiologists (*.*. and *.*.*.). The presence of LGE was evaluated across long-axis, short-axis, and four-chamber views. The myocardial strain analysis was based on manual delineation of endocardial and epicardial borders at end-diastole, excluding the papillary muscles and chordae. Then, the software automatically generated global myocardial strain parameters and corresponding “bull’s-eye” maps (Fig 2).

Inter- and intra-observer variability for strain measurements was assessed by comparing the analyses of two independent observers with at least 2-week interval between readings, thereby maintaining blinding to previous results. The coefficient of variation (CV) was calculated using the following formula: CV (%) = (s/X) × 100, where s represents the standard deviation and X indicates the mean of the measurements. Precision was expressed as the average %CV. If the CV was < 15%, the initial measurements were considered final.

### 2.3. Follow-up

Follow-up data were collected by reviewing hospital records, outpatient clinic visits, or telephone interviews conducted by a committee of radiologists who were blinded to the CMR findings. Duration of disease was defined as the time interval from the documented diagnosis of dilated cardiomyopathy to the baseline CMR examination (the first CMR performed after diagnostic confirmation). As baseline CMR was acquired as part of the initial diagnostic work-up, the timing of CMR relative to initiation and subsequent optimization of guideline-directed medical therapy was not predefined or standardized. Follow-up duration was defined as the interval from the initial CMR examination to the occurrence of an endpoint event or the last recorded patient contact. The primary endpoints were SCD, aborted SCD, and cardiac arrest secondary to ventricular fibrillation or sustained ventricular tachycardia. The secondary endpoint was a composite of all-cause mortality, heart failure-related rehospitalization, heart transplantation, and appropriate implantable cardioverter-defibrillator (ICD) therapy.

### 2.4. Statistical analysis

Statistical analyses were conducted using the Statistical Package for the Social Sciences software (version 26.0; IBM Corp., Armonk, NY, the USA) and R Studio (version 4.3.3). Continuous variables were presented as the mean ± standard deviation or the median with interquartile range (IQR), as appropriate. Meanwhile, categorical variables were expressed as the frequencies and percentages. Group comparisons for continuous variables were made using the independent Student’s *t*-test, one-way analysis of variance, or Mann–Whitney U test. The χ² test was used for categorical variables. The survival analysis included Kaplan–Meier curves with log-rank tests and univariate Cox regression to identify the risk factors of the primary and secondary outcomes. Significant variables from the univariate analysis and clinically relevant factors were included in the multivariate Cox models, with multicollinearity assessed using the variance inflation factor.

Four Cox models were developed and internally validated on complete-case data: model 1 (GLS), model 2 (LVEF), model 3 (GLS+LVEF), and model 4 (GLS+LVEF+age+NYHA). Model discrimination was evaluated using the Harrell’s C-index, reported as apparent and optimism-corrected (via stratified bootstrap, B = 1,000). Pairwise comparisons of ΔC used the same resamples with two-sided sign tests and Holm correction. A two-sided *P*-value of <0.05 indicated statistically significant differences. In parallel, hierarchical (block-entry) Cox models were used to test the incremental value of GLS beyond established clinical and conventional remodeling indices for both endpoints. We evaluated GLS on top of (i) Age + NYHA class (III/IV vs I/II) plus left atrial diameter (LAD), left ventricular mass index (LVMassi), or left ventricular end-diastolic volume index (LVEDVi), and (ii) logNT-proBNP plus LAD/LVMi/LVEDVi. The incremental value of GLS was assessed using likelihood-ratio tests comparing nested models; discrimination was reported using Harrell’s C-index.

## 3. Results

### 3.1. Characteristics of the patients and CMR parameters

**Fig 3:**
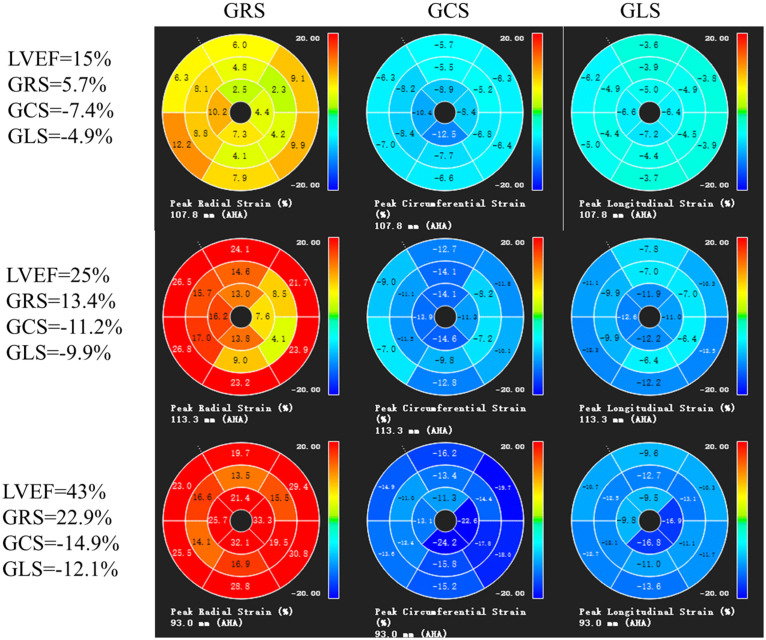
The “bull’s eye” diagram of left ventricular myocardial strain in patients with DCM with different LVEF.

The current study included 378 patients (76.5% men, mean age: 38.65 ± 14.20 years). At baseline, 40.2% of the patients were classified as having NYHA class III or higher disease. The mean LVEF was 24.61% ± 10.25%, with 68 patients having LVEF >35%, 175 with LVEF 20%–35%, and 135 with LVEF <20%. Patients with LVEF <20% had a significantly lower body surface area and resting systolic blood pressure, and a higher heart rate and log N-terminal pro–B-type natriuretic peptide (NT-proBNP) levels than those with LVEF >35% (all *P* < 0.005; Table 1). The intra- and inter-observer CVs for myocardial strain measurements were 5.54%–9.32% for global radial strain (GRS), 8.48%–9.72% for global circumferential strain (GCS), and 8.43%–8.95% for global longitudinal strain (GLS), indicating good reproducibility. Initial strain measurements were used for the final analysis. The median GRS, GCS, and GLS values were 12.04%, −11.40%, and −8.87%, respectively. Patients with an LVEF <20% had a significantly lower GRS and more impaired (i.e., less negative) GCS and GLS values than those with LVEF >35% and those with LVEF 20%–35% (all *P* < 0.001; Table 1 and Fig 3). During follow-up, the patients who experienced endpoint events had a shorter median follow-up time (58.47 months, IQR: 40.99–84.27 months) than those without events (60.23 months, IQR: 40.48–91.73 months).

**Table 1:** Baseline Characteristics for the 378 enrolled DCM patients.

	DCM(N = 378)	LVEF<20% (N = 135)	LVEF = 20–35%(N = 175)	LVEF>35%(N = 68)	*P* value
Age (years)	38.65 ± 14.20	39.53 ± 15.41	38.63 ± 13.99	36.94 ± 12.09	0.542
Male,n(%)	289(76.5)	105(77.8)	127(72.6)	57(83.8)	0.161
duration of disease(days)	4(1,12)	3(1,10)	6(1,17.5)*	5(1,20)	0.026
BSA(m^2^)	1.86(1.69,2.01)	1.84(1.68,1.98)	1.87(1.67,2.03)	1.93(1.74,2.07)*	0.077
Resting heart rate(beats/min)	75(65,88)	78(70,93)	75(64,87)*	68.5(58,76)*^#^	<0.001
Resting systolic blood pressure(mmHg)	118(105.75,130)	110(102,123)	120(109,130)*	117(110,130)*	0.001
Resting diastolic blood pressure(mmHg)	74(68,81)	71 (65,80)	77(68,83)	74(68.25,80)	0.248
NYHA functional class,n(%)					<0.001
I	26(6.9)	4(3.0)	13(7.4)	9(13.2)	
II	194(51.3)	44(32.6)	106(60.6)*	44(64.7)*	
III	125(33.1)	64(47.4)	47(26.9)*	14(20.6)*	
IV	33(8.7)	23(17.0)	9(5.1)*	1(1.5)*	
Atrial flutter or atrial fibrillation,n(%)	49(13)	19(14.1)	26(14.9)	4(5.9)	0.155
Nonsustained ventricular tachycardia,n(%)	27(7.1)	9(6.7)	14(8)	4(5.9)	0.897
Ventricular premature contraction,n(%)	78(20.6)	34(25.2)	33(18.9)	11(16.2)	0.252
LBBB,n(%)	26(6.9)	16(11.9)	8(4.6)*	2(2.9)*	0.023
Pulmonary hypertension,n(%)	30(7.9)	17(12.6)	11(6.3)	2(2.9)*	0.030
Mitral insufficiency,n(%)	107(28.3)	42(31.1)	44(25.1)	21(30.9)	0.447
Tricuspid insufficiency,n(%)	57(15.1)	23(17)	23(13.1)	11(16.2)	0.612
**Comorbidity**					
Diabetes,n(%)	34(9)	14(10.4)	12(6.9)	8(11.8)	0.382
Hyperlipidemia,n(%)	89(23.5)	33(24.4)	41(23.4)	15(22.1)	0.930
Hypertension,n(%)	105(27.8)	40(29.6)	48(27.4)	17(25)	0.778
Smoking history,n(%)	105(27.8)	50(37)	47(26.9)	8(11.8)*^#^	0.001
Perinatal period,n(%)	6(1.6)	3(2.2)	3(1.7)	0(0.0)	0.756
DCM family history,n(%)	12(3.2)	6(4.4)	4(2.3)	2(2.9)	0.494
SCD family history,n(%)	7(1.9)	1(0.7)	5(2.9)	1(1.5)	0.475
**Laboratory values**					
Log NT-proBNP	2.74(2.10,3.16)	3.03(2.63,3.40)	2.65(2.00,3.07)*	2.12(1.68,2.65)*^#^	<0.001
CK(U/L)	78(56,108.25)	84(57,139)	75(55,98)*	75(55.25,107.75)	0.028
Creatinine(μmol/L)	79(66,92)	79.87(67,95)	81(64,92)	75.05(61.25,86.58)	0.453
HCT(%)	43.7(40.38,46.4)	43.7(40.3,47)	43.3(40.3,46.4)	44.35(40.77,45.9)	0.631
HGB(g/L)	150(137,159)	151(138,162)	148(137,159)	150(131.25,156)	0.212
LVmassi(g/m²)	59.12(45.18,73.37)	69.11(54.47,85.59)	55.2(44.79,68.83)*	50.95(40.67,60.87)*^#^	<0.001
LVEDVi(mL/m²)	130.42(107.5,162.27)	163.10(135.12,205.6)	126.18(105.11,148.97)*	104.83(95.39,115.76)*^#^	<0.001
LVESV (mL)	183.32(132.89,246.93)	253.31(209.55,329.28)	168.2(136.92,209.53)*	115.99(101.28,133.36)*^#^	<0.001
LVCO (L/min)	4.22(3.27,5.28)	3.43(2.81,4.39)	4.48(3.56,5.41)*	5.07(4.25,6.21)*^#^	<0.001
LVSV (mL)	57.29(44.07,74.10)	41.76(33.8,52.94)	60.97(50.64,74.48)*	81.08(68.99,96.03)*^#^	<0.001
LVEDD (mm)	66.5(62,73)	72(66,80)	66(62,70)*	63(60,65)*^#^	<0.001
LAD (mm)	37(30,44)	42(35,49)	35(30,42)*	33(28,38)*^#^	<0.001
LV GRS (%)	12.04(8.94,16.51)	8.47(7.25,10.46)	12.7(10.64,16.13)*	19.86(17.39,23.98)*^#^	<0.001
LV GCS (%)	−11.40(−14.47,-8.22)	−7.76(−9.24,-6.6)	−12.16(−14.37,-10.74)*	−16.91(−18.94,-14.89)*^#^	<0.001
LV GLS (%)	−8.87(−11.04,-7.25)	−7.47(−8.48,-6.42)	−9.59(−11.25,-7.71)*	−11.48(−13.53,-9.89)*^#^	<0.001
ACE inhibitors/ ARB,n(%)	226(59.8)	74(54.8)	110 (62.9)	42(61.8)	0.335
Beta-blocker,n(%)	280(74.1)	100(74.1)	134(76.6)	46(67.6)	0.362
Spironolactone,n(%)	269(71.2)	100(74.1)	124(70.9)	45(66.2)	0.499
Diuretic,n(%)	185(48.9)	90(66.7)	83(47.4)	12(17.6)	<0.001
Warfarin,n(%)	21(5.6)	9(6.7)	10(5.7)	2(2.9)	0.579
Aspirin,n(%)	97(25.7)	39(28.9)	47(26.9)	11(16.2)	0.130
Digoxin,n(%)	155(41.0)	80(59.3)	59(33.7)	16(23.5)	<0.001
Statins,n(%)	32(8.5)	16(11.9)	11(6.3)	5(7.4)	0.204
Amiodarone,n(%)	16(4.2)	2(1.5)	14(8.0)	0(0.0)	0.004

Values are n (%), median (interquartile range), or mean±SD.

LVEF = LV ejection fraction; BSA = body surface area; NYHA = New York Heart Association; LBBB = left bundle branch block; DCM = dilated cardiomyopathy; SCD = sudden cardiac death; NT-proBNP = N-terminal pro–B-type natriuretic peptide(log-transformed for inclusion in models); CK = creatine kinase; HCT = hematocrit; HGB = hemoglobin; LVmassi = LV mass index; LVEDVi = LV end-diastolic volume index; LVESV = LV end systolic volume; LVCO = LV cardiac output; LVSV = LV stroke volume; LVEDD = LV end diastolic dimension; LAD = left atrial diameter; GRS = global radial strain; GCS = global circumferential strain; GLS = global longitudinal strain.

* indicates statistical significance compared to LVEF < 20%; ^#^ indicates statistical significance compared to LVEF = 20–35%.

### 3.2. Association between CMR, clinical variables and the primary endpoints

During a median follow-up of 59.78 (IQR: 40.70–89.86) months, 35 (9.26%) patients reached the primary endpoints (29 SCD/aborted SCD, 6 VA events). According to the univariate Cox regression analysis, the significant predictors of the primary endpoints included age, body surface area, heart rate, history of left bundle branch block, log-transformed NT-proBNP levels, NYHA functional class III/IV, and creatine kinase levels (all *P* < 0.05; Table 2). In the overall cohort, GRS (hazard ratio [HR] = 0.81, 95% confidence interval [CI]: 0.74–0.90, *P* < 0.001), GCS (HR = 1.29, 95% CI: 1.15–1.45, *P* < 0.001), and GLS (HR = 1.49, 95% CI: 1.23–1.80, *P* < 0.001) were strongly associated with the primary endpoints. These associations remained significant in the LVEF-based subgroup analyses (including LVEF <35% and <20%; Table 3). The receiver operating characteristic curve analysis revealed that the optimal cutoff values for predicting the primary endpoints were as follows: 11.37% for GRS, −9.93% for GCS, and −9.61% for GLS, with the corresponding area under the curve, sensitivity, and specificity values detailed in Table 4. Based on the multivariate Cox regression analysis, GLS (HR = 1.29, 95% CI: 1.04–1.59, *P* = 0.019), LVEF (HR = 0.93, 95% CI: 0.89–0.98, *P* = 0.004), and age (HR = 1.03, 95% CI: 1.01–1.06, *P* = 0.035) were independent predictors of the primary endpoints in the overall cohort. These associations remained significant in patients with LVEF <35%. However, only GLS (HR = 1.37, 95% CI: 1.01–1.85, *P* = 0.041) retained its prognostic value in the LVEF <20% subgroup (Table 3).

**Table 2:** The univariate Cox regression analyses for primary endpoints and secondary endpoints.

	Primary endpoints (n = 35)		Secondary endpoints (n = 72)	
variable	HR (95%CI)	*P* value	HR (95%CI)	*P* value
Age	1.03 (1.01-1.06)	0.006	1.02(1.01-1.04)	0.005
male	0.99(0.43-2.27)	0.979	1.67(1.01-2.77)	0.045
duration of disease	1.00 (1.00-1.01)	0.228	1.00(1.00-1.01)	0.363
BSA	0.10(0.02-0.41)	0.002	0.22(0.08-0.59)	0.002
Resting heart rate	1.02(1.00-1.04)	0.008	1.01(1.00-1.03)	0.088
Resting systolic blood pressure	0.98(0.96-1.00)	0.079	0.99(0.98-1.00)	0.130
Resting diastolic blood pressure	0.98(0.95-1.01)	0.130	1.00 (0.98-1.01)	0.651
NYHA functional class				
I	1 [Reference]	NA	1 [Reference]	NA
II	0.67(0.15-3.01)	0.602	1.07(0.33-3.55)	0.907
III	1.76(0.40-7.70)	0.456	2.63(0.80-8.58)	0.110
IV	2.40(0.48-12.00)	0.288	2.59(0.70-9.59)	0.156
Atrial flutter or atrial fibrillation	0.58(0.18-1.89)	0.364	0.67(0.31-1.45)	0.307
Short array ventricular tachycardia	1.92(0.68-5.43)	0.222	1.30(0.56-2.99)	0.543
Ventricular premature contraction	1.35(0.63-2.89)	0.438	1.49(0.89-2.50)	0.129
LBBB	2.97(1.15-7.67)	0.024	2.17(1.04-4.53)	0.039
Pulmonary hypertension	2.70(0.94-7.76)	0.065	1.43(0.57-3.57)	0.444
Mitral insufficiency	1.23(0.55-2.75)	0.614	1.73(1.03-2.89)	0.037
Mricuspid insufficiency	1.53(0.58-4.02)	0.386	1.06(0.50-2.34)	0.883
**Comorbidity**				
Diabetes	1.64(0.64-4.23)	0.307	1.45(0.72-2.92)	0.297
Hyperlipidemia	1.05(0.48-2.32)	0.898	1.24(0.73-2.10)	0.420
Hypertension	1.55(0.77-3.12)	0.225	0.95(0.55-1.62)	0.840
Smoking history	1.38(0.69-2.76)	0.356	0.98(0.59-1.64)	0.947
DCM family history	2.15(0.51-8.97)	0.296	2.01(0.73-5.53)	0.174
SCD family history	1.08(0.15-7.90)	0.943	1.11(0.27-4.52)	0.890
**Laboratory values**				
Log NT-proBNP	2.39(1.32-4.32)	0.004	2.80(1.83-4.27)	<0.001
Creatine kinase (CK)	1.00 (1.00-1.01)	<0.001	1.00(1.00-1.01)	<0.001
Creatinine	1.01(1.00-1.02)	0.224	0.99(0.98-1.00)	0.181
HCT	0.98(0.92-1.05)	0.596	1.00(0.96-1.04)	0.954
HGB	1.00(0.98-1.02)	0.884	1.00(0.99-1.01)	0.749
**CMR parameters**				
LVmassi	1.02 (1.01–1.04)	<0.001	1.02 (1.01–1.03)	<0.001
LVEDVi	1.01 (1.01–1.01)	<0.001	1.01 (1.01–1.01)	<0.001
LVESV	1.00(1.00-1.00)	<0.001	1.00(1.00-1.00)	<0.001
LVEF	0.91(0.87-0.95)	<0.001	0.94(0.92-0.97)	<0.001
LVCO	0.79(0.61-1.02)	0.066	0.81(0.68-0.96)	0.017
LVSV	0.97(0.96-0.99)	0.001	0.98(0.97-0.99)	<0.001
LVEDD	1.08(1.05-1.11)	<0.001	1.05(1.02-1.07)	<0.001
LAD	1.04(1.01-1.07)	0.009	1.04(1.02-1.06)	<0.001

LVEF = LV ejection fraction; BSA = body surface area; NYHA = New York Heart Association; LBBB = left bundle branch block; DCM = dilated cardiomyopathy; SCD = sudden cardiac death; NT-proBNP = N-terminal pro–B-type natriuretic peptide(log-transformed for inclusion in models); CK = creatine kinase; HCT = hematocrit; HGB = hemoglobin; LVmassi = LV mass index; LVEDVi = LV end-diastolic volume index; LVESV = LV end systolic volume; LVCO = LV cardiac output; LVSV = LV stroke volume; LVEDD = LV end diastolic dimension; LAD = left atrial diameter; GRS = global radial strain; GCS = global circumferential strain; GLS = global longitudinal strain.

**Table 3:** Univariate and multivariate cox regression for primary endpoints.

	Any LVEF		LVEF＜35%		LVEF＜20%	
	HR (95% CI)	P value	HR (95% CI)	P value	HR (95% CI)	P value
**univariable analyses**						
GRS	0.81(0.74-0.90)	<0.001	0.83(0.74-0.93)	0.001	0.99(0.86-1.14)	0.895
GCS	1.29(1.15-1.45)	<0.001	1.28(1.12-1.47)	<0.001	1.05(0.82-1.34)	0.694
GLS	1.49(1.23-1.80)	<0.001	1.41(1.16-1.72)	0.001	1.37(1.01-1.85)	0.041
LVEF	0.91(0.87-0.95)	<0.001	0.91(0.87-0.95)	<0.001	1.05(0.95-1.17)	0.347
Age	1.03 (1.01-1.06)	0.006	1.04(1.01-1.06)	0.005	1.02(1.00-1.05)	0.103
NYHA class III or IV (vs I or II)	2.70(1.37-5.34)	0.004	2.46(1.23-4.94)	0.011	1.54(0.67-3.52)	0.309
**multivariable analyses**						
GLS	1.29(1.04-1.59)	0.019	1.24(1.01-1.54)	0.044	1.37(1.01-1.85)	0.041
LVEF	0.94(0.89-0.98)	0.005	0.93(0.88-0.98)	0.007	–	–
Age	1.03(1.01-1.06)	0.008	1.03(1.01-1.06)	0.008	–	–
NYHA class III or IV (vs I or II)	–	–	–	–	–	–

LVEF = LV ejection fraction; BSA = body surface area; NYHA = New York Heart Association; LBBB = left bundle branch block; DCM = dilated cardiomyopathy; SCD = sudden cardiac death; NT-proBNP = N-terminal pro–B-type natriuretic peptide(log-transformed for inclusion in models); CK = creatine kinase; HCT = hematocrit; HGB = hemoglobin; LVmassi = LV mass index; LVEDVi = LV end-diastolic volume index; LVESV = LV end systolic volume; LVCO = LV cardiac output; LVSV = LV stroke volume; LVEDD = LV end diastolic dimension; LAD = left atrial diameter; GRS = global radial strain; GCS = global circumferential strain; GLS = global longitudinal strain.

**Table 4:** The ROC curve analysis for primary endpoints in the LVEF subgroup of patients with DCM.

	Cutoff	AUC	Sensitivity (%)	Specificity (%)
Any LVEF				
GRS (%)	11.37	0.720	59.5	80.0
GCS (%)	−9.925	0.744	77.1	64.4
GLS (%)	−9.605	0.703	88.6	43.1
LVEF＜35%				
GRS (%)	11.37	0.668	49.6	82.4
GCS (%)	−8.85	0.708	67.6	68.5
GLS (%)	−7.84	0.668	64.7	62.0
LVEF＜20%				
GRS (%)	7.84	0.502	63.9	51.9
GCS (%)	−6.955	0.580	51.9	66.7
GLS (%)	−5.68	0.578	29.6	92.6

LVEF = LV ejection fraction; BSA = body surface area; NYHA = New York Heart Association; LBBB = left bundle branch block; DCM = dilated cardiomyopathy; SCD = sudden cardiac death; NT-proBNP = N-terminal pro–B-type natriuretic peptide(log-transformed for inclusion in models); CK = creatine kinase; HCT = hematocrit; HGB = hemoglobin; LVmassi = LV mass index; LVEDVi = LV end-diastolic volume index; LVESV = LV end systolic volume; LVCO = LV cardiac output; LVSV = LV stroke volume; LVEDD = LV end diastolic dimension; LAD = left atrial diameter; GRS = global radial strain; GCS = global circumferential strain; GLS = global longitudinal strain.

In additional hierarchical Cox analyses, GLS remained independently associated with the primary endpoint and significantly improved model fit when added to models including Age+NYHA plus LAD/LVmassi/LVEDVi, as well as models including log NT-proBNP plus LAD/LVmassi/LVEDVi (all likelihood-ratio test *P* < 0.001; S1 and S2 Table in S1 File).

### 3.3. Association between CMR, clinical variables and the secondary endpoint

During follow-up, 72 patients reached the composite secondary endpoint (n = 29 all-cause deaths; n = 33, heart failure-related hospitalizations; n = 2, heart transplants; and n = 8, appropriate ICD therapies). The univariate Cox regression analysis showed that the significant predictors of the secondary endpoint included age, male sex, left bundle branch block, NYHA functional class III/IV, high log-transformed NT-proBNP levels, and the presence of mitral insufficiency. In addition, myocardial strain parameters including GRS (HR = 0.89, 95% CI: 0.85–0.94, *P* < 0.001), GCS (HR = 1.16, 95% CI: 1.09–1.24, *P* < 0.001), and GLS (HR = 1.25, 95% CI: 1.13–1.39, *P* < 0.001) were significantly associated with the composite outcome. In the multivariate analysis, after adjusting for age, LVEF, and NYHA functional class III/IV (vs. I/II), only GLS remained an independent predictor of the secondary endpoint. In particular, each unit increase in GLS was associated with a 1.14-fold higher risk (HR = 1.14, 95% CI: 1.01–1.29, *P* = 0.037; Table 5).

**Table 5:** Univariate and multivariate cox regression for secondary endpoints.

	Any LVEF	
	HR (95% CI)	P value
**univariable analyses**		
GRS	0.89(0.85-0.94)	<0.001
GCS	1.16(1.09-1.24)	<0.001
GLS	1.25(1.13-1.39)	<0.001
NYHA class III or IV (vs I or II)	2.46(1.53-3.93)	<0.001
**multivariable analyses**		
GLS	1.14(1.01-1.29)	0.037
LVEF	0.96(0.93-0.99)	0.007
Age	1.02(1.01-1.04)	0.009
NYHA class III or IV (vs I or II)	–	–

Abbreviations as Table 1.

A similar incremental value of GLS was observed for the secondary endpoint across all hierarchical models (all likelihood-ratio test *P* ≤ 0.008; S3 and S4 Table in S1 File).

### 3.4. Novel prediction models combining strain and LVEF strata

Based on the strong association between myocardial strain and adverse outcomes, we evaluated different LVEF stratification schemes for discriminating the risk of SCD, aborted SCD, or VA in this cohort, and assessed predictive models combining strain with LVEF strata.

**Fig 4:**
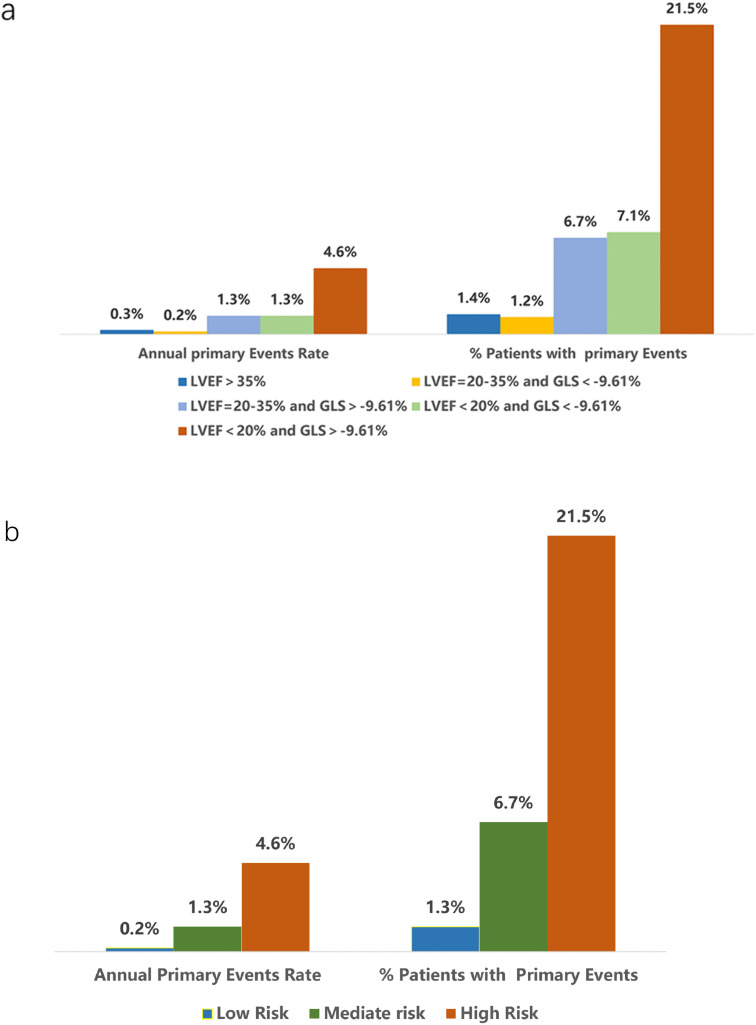
The bar graph of annual primary events rate by different LVEF groups combined with strain measurements (a). **The bar graph of annual primary events rate among low-risk, intermediate-risk, and high-risk groups (b)**.

According to a previous research, an LVEF of 35% is the recognized threshold in major clinical guidelines for considering primary prevention ICD therapy. Conversely, an LVEF of 20% indicates extremely severe ventricular systolic dysfunction, and it is consistently associated with a significantly elevated risk of adverse clinical events [20,21]. Our study revealed that the incidence of the primary endpoint was low in patients with an LVEF of >35% (n = 1, 1.5%; annual rate: 0.3%). However, it was substantially high in the LVEF <20% subgroup (n = 27, 20.0%; annual rate: 5.9%), with an intermediate event rate observed in the LVEF 20%–35% group (n = 7, 4.0%; annual rate: 1.5%). Based on these findings, three LVEF strata were defined as follows: > 35% (n = 68), 20%–35% (n = 175), and <20% (n = 135). A significant stepwise increase in the incidence of SCD, aborted SCD, or VA was observed across the three LVEF categories (*P* < 0.001; Fig 4a). To improve risk discrimination, a predictive model combining the optimal cutoff value of GLS with LVEF stratification was developed.

**Fig 5:**
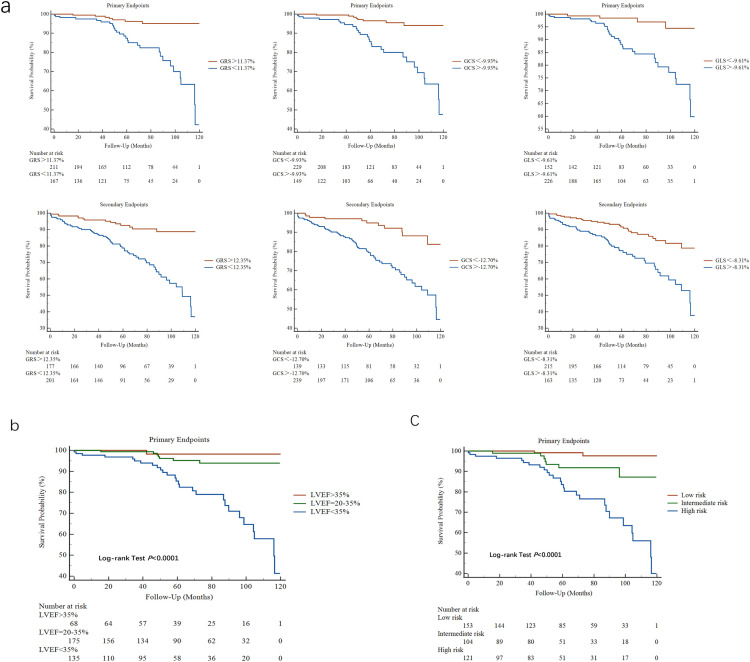
Prognostic stratification using ROC-derived cutoffs: Kaplan-meier analyses of survival by LVEF, strain, and risk groups. Using the cut off values obtained from ROC curve analysis, Kaplan-Meier curves were constructed for each LVEF subgroup **(a)**. Kaplan-Meier curve analysis of survival free from primary endpoints, incorporating both stratification by LVEF groups and assessment of strain measurements **(b)**. Kaplan-Meier curve analysis of survival free from primary endpoints among low-risk, intermediate-risk, and high-risk groups **(c)**.

Based on this approach, the patients were stratified into three risk groups: low-risk (LVEF >35% or LVEF 20%–35% with GLS <−9.61%; annual event rate: 0.2%), intermediate-risk (LVEF 20%–35% with GLS ≥ −9.61% or LVEF <20% with GLS <−9.61%; annual event rate: 1.3%), and high-risk (LVEF <20% with GLS ≥ −9.61%; annual event rate: 4.6%) (Fig 4b). The Kaplan–Meier analysis showed a significantly reduced event-free survival rate across these groups (Fig 5a–c). Compared with the low-risk group, the patients in the intermediate- and high-risk groups had progressively increased risks of the primary endpoints, with HRs of 5.45 (95% CI: 1.13–26.26; *P* = 0.035) and 20.33 (95% CI: 4.82–85.72; *P* < 0.001), respectively. In addition, the high-risk group had a significantly higher adjusted risk of SCD or aborted SCD than the intermediate-risk group (HR = 4.11, 95% CI: 1.69–9.99, *P* = 0.002).

To evaluate performance, the following three schemes were compared: (1) a GLS cutoff of −9.61% (model 1), (2) LVEF strata (>35%, 20%–35%, < 20%; model 2), and (3) a combined risk-group classification using both markers (model 3), with the addition of age and NYHA to create model 4. Model 3 was more effective in discriminating the primary endpoint than models 1 or 2 (C-index = 0.756 vs 0.681 and 0.714; *P* < 0.001). Adding age and NYHA classification further improved discrimination (model 4: apparent C-index = 0.801, 95% CI: 0.729–0.873) and outperformed model 3 (0.801 vs 0.756; *P* = 0.010). Model 4 (0.785, 95% CI: 0.719–0.858) had the highest optimism-corrected C-indices, followed by model 3 (0.754, 95% CI: 0.689–0.822), model 2 (0.711, 95% CI: 0.640–0.791), and model 1 (0.681, 95% CI: 0.636–0.734). Paired bootstrap comparisons (B = 1,000) confirmed that model 3 outperformed models 1 and 2 after correction (Holm-adjusted *P* = 0.048). However, the advantage of model 4 over model 3 did not reach statistical significance, even though the corrected C-index was numerically higher. S5 and S6 Table in S1 File show further details.

## 4. Discussion

In this multicenter cohort of patients with DCM who did not exhibit LGE on cardiac MRI, GLS had a substantial prognostic value beyond conventional structural and clinical markers. It remained an independent predictor of SCD-related events after controlling for LVEF, age, and NYHA classification, thereby emphasizing its sensitivity in detecting early myocardial dysfunction that may not manifest as volumetric decline or overt fibrosis. When incorporated into LVEF-based models, GLS significantly improved discriminative accuracy, and these enhancements remained consistent after bootstrap internal validation. Notably, among patients with severely impaired systolic function (LVEF <20%), GLS was the only independent risk marker. This finding underscores its unique value in identifying high-risk individuals in a group where traditional metrics provide limited stratification.

Previous literature has established the prognostic relevance of mid-wall fibrosis on LGE, especially for arrhythmic events and SCD in nonischemic DCM [4,22–24].However, some patients do not present with visible LGE despite having significant arrhythmic risk [3,5], thereby creating an important clinical blind spot. Previous studies have established the prognostic value of CMR feature tracking, including GLS. However, these analyses typically included mixed cohorts of LGE-positive and LGE-negative patients. Therefore, it is challenging to draw conclusions about the LGE-negative subgroup [25,26]. Our study directly addresses this gap by evaluating a relatively large, exclusively LGE-negative population, thereby offering a clearer understanding of residual risk when fibrosis cannot be detected on standard imaging. Consistent with earlier observations indicating that longitudinal dysfunction can precede circumferential impairment, we found that GLS—but not GCS—was associated with adverse outcomes, which is in accordance with previous data showing that GCS is less prognostically significant in LGE-negative DCM [6,27–29].

These findings can be explained by the distinct vulnerability of subendocardial longitudinal fibers, which are disproportionately susceptible to early interstitial fibrosis, microvascular dysfunction, inflammation, and cytoskeletal alterations—which are pathophysiologic processes commonly observed in patients with DCM even in the absence of macroscopic scar [30–32]. Such microstructural abnormalities may significantly impair longitudinal deformation, thereby contributing to electrical instability and arrhythmogenesis. This mechanism offers a compelling explanation for why GLS continues to predict malignant arrhythmic events even if traditional structural measurement parameters, such as LVEF, fail to completely identify the underlying risk. The strong association between GLS and adverse outcomes in patients with LVEF <20% further underscores its role in detecting disease severity and arrhythmic vulnerability that are not captured by global systolic function alone [33,34].

By integrating GLS cutoff values with established LVEF categories, this study developed a practical multiparametric risk stratification system that clearly distinguished low-, intermediate-, and high-risk groups using Kaplan–Meier survival curves. This approach may be valuable for LGE-negative individuals who often fall into a management “gray zone” regarding eligibility for ICD implantation or the need for intensified rhythm monitoring. Our findings support the notions that arrhythmic risk in nonischemic DCM cannot be fully identified by LVEF alone and that incorporating additional functional biomarkers may facilitate more individualized therapeutic decision-making, which is consistent with newer conceptual frameworks in DCM management [3,35,36]. Our study did not directly evaluate treatment modification based on GLS. Nevertheless, the observed stratification patterns indicated that GLS may help refine follow-up strategies, optimize timing for electrophysiological assessment, or contribute to shared decision-making regarding device therapy, particularly in intermediate-risk patients.

Considering that strain parameters predominantly capture functional impairment, combining GLS with tissue-level markers, such as T1 mapping and extracellular volume, may offer further incremental value. T1 mapping has a strong prognostic performance even among patients without replacement fibrosis [37–39]. However, heterogeneity in acquisition protocols, scanner platforms, and cutoff values currently limits its widespread adoption. As emphasized in recent methodological analyses, harmonized multicenter studies and validation of standardized thresholds will be essential to integrating T1-based diffuse fibrosis assessment into clinical risk models. Such integration may eventually lead to the development of a more comprehensive imaging-based risk classification system that simultaneously captures structural, functional, and tissue-level abnormalities [40].

The current study has several limitations. First, although consecutive patients from multiple centers were included, the retrospective design may introduce selection and information bias. The number of primary endpoint events was relatively modest, which constrained the complexity of multivariable models; therefore, we prespecified parsimonious models and applied bootstrap internal validation to reduce the risk of overfitting. Second, outcomes were analyzed using time-to-first event, and competing-risk analyses were not performed. Third, CMR-FT strain analysis was conducted using a single vendor-specific platform, and reproducibility assessments relied on CV rather than intraclass correlation, which might have restricted generalizability across imaging systems.

Fourth, baseline CMR was performed as part of the initial diagnostic work-up during the index hospitalization; therefore, the timing of CMR relative to initiation and subsequent optimization of guideline-directed medical therapy was not predefined or standardized across patients. In addition, although baseline medication use was recorded, treatment initiation, dose titration, adherence, and therapy modifications during follow-up (as well as variations in follow-up intensity) were not systematically captured and could have influenced clinical outcomes. Fifth, advanced CMR tissue characterization parameters (e.g., native T1 and extracellular volume mapping), which may further refine risk stratification in LGE-negative DCM, were not available. In addition, due to the retrospective nature of the study and heterogeneity in acquisition protocols, right ventricular volume/function and LV sphericity metrics were not consistently available; thus, their independent prognostic value and incremental contribution could not be evaluated. Finally, high-sensitivity cardiac troponin was not routinely measured in the earlier period of our cohort; therefore, creatine kinase (CK) was used as a surrogate marker. In our inpatient cohort, elevated CK may reflect acute myocardial injury and/or clinical decompensation at the index hospitalization, which may partly explain its prognostic association.

In conclusion, GLS has a robust incremental prognostic value beyond LVEF in LGE-negative DCM. Moreover, it enhances risk stratification when combined with age, NYHA classification, and LVEF. These findings underscore the clinical value of myocardial deformation analysis in a subgroup of patients where conventional imaging markers may be insufficient, thereby providing a pathway toward a more precise and individualized arrhythmic risk assessment. Nevertheless, prospective multicenter studies incorporating advanced tissue characterization and evaluating GLS-guided management strategies should be conducted to further define the role of GLS in routine clinical practice.

## Supporting information

S1 File Supplementary Tables (including Tables S1–S6). (DOCX)

## References

[1] Arbelo E, Protonotarios A, Gimeno JR, Arbustini E, Barriales-Villa R, Basso C, et al. 2023 ESC Guidelines for the management of cardiomyopathies. Eur Heart J. 2023;44(37):3503–626. doi: 10.1093/eurheartj/ehad194 37622657

[2] Maron BJ, Towbin JA, Thiene G, Antzelevitch C, Corrado D, Arnett D, et al. Contemporary definitions and classification of the cardiomyopathies: an American Heart Association Scientific Statement from the Council on Clinical Cardiology, Heart Failure and Transplantation Committee; Quality of Care and Outcomes Research and Functional Genomics and Translational Biology Interdisciplinary Working Groups; and Council on Epidemiology and Prevention. Circulation. 2006;113(14):1807–16. doi: 10.1161/CIRCULATIONAHA.106.174287 16567565

[3] Akhtar M, Elliott PM. Risk stratification for sudden cardiac death in non-ischaemic dilated cardiomyopathy. Curr Cardiol Rep. 2019;21(12):155. doi: 10.1007/s11886-019-1236-3 31768884 PMC6877704

[4] Becker MAJ, Cornel JH, van de Ven PM, van Rossum AC, Allaart CP, Germans T. The prognostic value of late gadolinium-enhanced cardiac magnetic resonance imaging in nonischemic dilated cardiomyopathy: A review and meta-analysis. JACC Cardiovasc Imaging. 2018;11(9):1274–84. doi: 10.1016/j.jcmg.2018.03.006 29680351

[5] Merlo M, Cannatà A, Pio Loco C, Stolfo D, Barbati G, Artico J, et al. Contemporary survival trends and aetiological characterization in non-ischaemic dilated cardiomyopathy. Eur J Heart Fail. 2020;22(7):1111–21. doi: 10.1002/ejhf.1914 32452075

[6] Li S, Wang Y, Yang W, Zhou D, Zhuang B, Xu J, et al. Cardiac MRI risk stratification for dilated cardiomyopathy with left ventricular ejection fraction of 35% or higher. Radiology. 2023;306(3):e213059. doi: 10.1148/radiol.213059 36318031 PMC9968772

[7] Dawson DK, Hawlisch K, Prescott G, Roussin I, Di Pietro E, Deac M, et al. Prognostic role of CMR in patients presenting with ventricular arrhythmias. JACC Cardiovasc Imaging. 2013;6(3):335–44. doi: 10.1016/j.jcmg.2012.09.012 23433931

[8] Alba AC, Gaztañaga J, Foroutan F, Thavendiranathan P, Merlo M, Alonso-Rodriguez D, et al. Prognostic value of late gadolinium enhancement for the prediction of cardiovascular outcomes in dilated cardiomyopathy: An international, multi-institutional study of the MINICOR Group. Circ Cardiovasc Imaging. 2020;13(4):e010105. doi: 10.1161/CIRCIMAGING.119.010105 32312112

[9] Gulati A, Jabbour A, Ismail TF, Guha K, Khwaja J, Raza S, et al. Association of fibrosis with mortality and sudden cardiac death in patients with nonischemic dilated cardiomyopathy. JAMA. 2013;309(9):896–908. doi: 10.1001/jama.2013.1363 23462786

[10] Di Marco A, Anguera I, Schmitt M, et al. Late gadolinium enhancement and the risk for ventricular arrhythmias or sudden death in dilated cardiomyopathy: Systematic review and meta-analysis. JACC Heart Fail. 2017;5(1):28–38. doi: 10.1016/j.jchf.2016.09.017 28017348

[11] Tang HS, Kwan CT, He J, Ng PP, Hai SHJ, Kwok FYJ, et al. Prognostic Utility of Cardiac MRI myocardial strain parameters in patients with ischemic and nonischemic dilated cardiomyopathy: A multicenter study. AJR Am J Roentgenol. 2023;220(4):524–38. doi: 10.2214/AJR.22.28415 36321987

[12] McDonagh TA, Metra M, Adamo M, Gardner RS, Baumbach A, Böhm M, et al. 2021 ESC Guidelines for the diagnosis and treatment of acute and chronic heart failure. Eur Heart J. 2021;42(36):3599–726. doi: 10.1093/eurheartj/ehab368 34447992

[13] Stecker EC, Vickers C, Waltz J, Socoteanu C, John BT, Mariani R, et al. Population-based analysis of sudden cardiac death with and without left ventricular systolic dysfunction: Two-year findings from the Oregon Sudden Unexpected Death Study. J Am Coll Cardiol. 2006;47(6):1161–6. doi: 10.1016/j.jacc.2005.11.045 16545646

[14] Di Marco A, Brown PF, Bradley J, Nucifora G, Claver E, de Frutos F, et al. Improved risk stratification for ventricular arrhythmias and sudden death in patients with nonischemic dilated cardiomyopathy. J Am Coll Cardiol. 2021;77(23):2890–905. doi: 10.1016/j.jacc.2021.04.030 34112317

[15] Romano S, Judd RM, Kim RJ, Kim HW, Klem I, Heitner J, et al. Association of feature-tracking cardiac magnetic resonance imaging left ventricular global longitudinal strain with all-cause mortality in patients with reduced left ventricular ejection fraction. Circulation. 2017;135(23):2313–5. doi: 10.1161/CIRCULATIONAHA.117.027740 28584033 PMC5494997

[16] Csecs I, Pashakhanloo F, Paskavitz A, Jang J, Al-Otaibi T, Neisius U, et al. Association between left ventricular mechanical deformation and myocardial fibrosis in nonischemic cardiomyopathy. J Am Heart Assoc. 2020;9(19):e016797. doi: 10.1161/JAHA.120.016797 33006296 PMC7792406

[17] Ochs A, Riffel J, Ochs MM, Arenja N, Fritz T, Galuschky C, et al. Myocardial mechanics in dilated cardiomyopathy: prognostic value of left ventricular torsion and strain. J Cardiovasc Magn Reson. 2021;23(1):136. doi: 10.1186/s12968-021-00829-x 34852822 PMC8638178

[18] Buss SJ, Breuninger K, Lehrke S, Voss A, Galuschky C, Lossnitzer D, et al. Assessment of myocardial deformation with cardiac magnetic resonance strain imaging improves risk stratification in patients with dilated cardiomyopathy. Eur Heart J Cardiovasc Imaging. 2015;16(3):307–15. doi: 10.1093/ehjci/jeu181 25246506

[19] Sengeløv M, Jørgensen PG, Jensen JS, Bruun NE, Olsen FJ, Fritz-Hansen T, et al. Global longitudinal strain is a superior predictor of all-cause mortality in heart failure with reduced ejection fraction. JACC Cardiovasc Imaging. 2015;8(12):1351–9. doi: 10.1016/j.jcmg.2015.07.013 26577264

[20] Al-Jefairi N, Burri H. Relevance of guideline-based ICD indications to clinical practice. Indian Heart J. 2014;66 Suppl 1(Suppl 1):S82-7. doi: 10.1016/j.ihj.2013.11.006 24568834 PMC4237301

[21] Cheng S, Deng Y, Huang H, Liu X, Yu Y, Chen X, et al. Prognostic implications of left ventricular ejection fraction and left ventricular end-diastolic diameter on clinical outcomes in patients with ICD. J Cardiovasc Dev Dis. 2022;9(12):421. doi: 10.3390/jcdd9120421 36547418 PMC9782887

[22] Halliday BP, Gulati A, Ali A, Guha K, Newsome S, Arzanauskaite M, et al. Association between midwall late gadolinium enhancement and sudden cardiac death in patients with dilated cardiomyopathy and mild and moderate left ventricular systolic dysfunction. Circulation. 2017;135(22):2106–15. doi: 10.1161/CIRCULATIONAHA.116.026910 28351901 PMC5444425

[23] Halliday BP, Baksi AJ, Gulati A, et al. Outcome in dilated cardiomyopathy related to the extent, location, and pattern of late gadolinium enhancement. JACC Cardiovasc Imaging. 2019;12(8 Pt 2):1645–55. doi: 10.1016/j.jcmg.2018.07.015 30219397 PMC6682609

[24] Wang J, Yang F, Wan K, Mui D, Han Y, Chen Y. Left ventricular midwall fibrosis as a predictor of sudden cardiac death in non-ischaemic dilated cardiomyopathy: A meta-analysis. ESC Heart Fail. 2020;7(5):2184–92. doi: 10.1002/ehf2.12865 32603034 PMC7524301

[25] He J, Yang W, Wu W, Li S, Yin G, Zhuang B, et al. Early diastolic longitudinal strain rate at MRI and outcomes in heart failure with preserved ejection fraction. Radiology. 2021;301(3):582–92. doi: 10.1148/radiol.2021210188 34519577 PMC8630598

[26] Jung IH, Park JH, Lee JA, Kim GS, Lee HY, Byun YS, et al. Left ventricular global longitudinal strain as a predictor for left ventricular reverse remodeling in dilated cardiomyopathy. J Cardiovasc Imaging. 2020;28(2):137–49. doi: 10.4250/jcvi.2019.0111 32233166 PMC7114450

[27] Shu S-L, Wang J, Wang C, Zhu F, Jia Y-X, Zhang L, et al. Prognostic value of feature-tracking circumferential strain in dilated cardiomyopathy patients with severely reduced ejection fraction incremental to late gadolinium enhancement. Curr Med Sci. 2021;41(1):158–66. doi: 10.1007/s11596-021-2331-4 33582921

[28] Moon JC, Messroghli DR, Kellman P, Piechnik SK, Robson MD, Ugander M, et al. Myocardial T1 mapping and extracellular volume quantification: A Society for Cardiovascular Magnetic Resonance (SCMR) and CMR Working Group of the European Society of Cardiology consensus statement. J Cardiovasc Magn Reson. 2013;15(1):92. doi: 10.1186/1532-429X-15-92 24124732 PMC3854458

[29] Chimura M, Onishi T, Tsukishiro Y, Sawada T, Kiuchi K, Shimane A, et al. Longitudinal strain combined with delayed-enhancement magnetic resonance improves risk stratification in patients with dilated cardiomyopathy. Heart. 2017;103(9):679–86. doi: 10.1136/heartjnl-2016-309746 27799316

[30] Merlo M, Cannatà A, Gobbo M, Stolfo D, Elliott PM, Sinagra G. Evolving concepts in dilated cardiomyopathy. Eur J Heart Fail. 2018;20(2):228–39. doi: 10.1002/ejhf.1103 29271570

[31] Xu Y, Lin J, Liang Y, Wan K, Li W, Wang J, et al. Prognostic value of left ventricular remodelling index in idiopathic dilated cardiomyopathy. Eur Heart J Cardiovasc Imaging. 2021;22(10):1197–207. doi: 10.1093/ehjci/jeaa144 32658979

[32] Yu Y, Yu S, Tang X, Ren H, Li S, Zou Q, et al. Evaluation of left ventricular strain in patients with dilated cardiomyopathy. J Int Med Res. 2017;45(6):2092–100. doi: 10.1177/0300060517712164 28587541 PMC5805211

[33] Liu T, Gao Y, Wang H, Zhou Z, Wang R, Chang S-S, et al. Association between right ventricular strain and outcomes in patients with dilated cardiomyopathy. Heart. 2021;107(15):1233–9. doi: 10.1136/heartjnl-2020-317949 33139324 PMC8292584

[34] Romano S, Judd RM, Kim RJ, et al. Feature-tracking global longitudinal strain predicts death in a multicenter population of patients with ischemic and nonischemic dilated cardiomyopathy incremental to ejection fraction and late gadolinium enhancement. JACC Cardiovasc Imaging. 2018;11(10):1419–29. doi: 10.1016/j.jcmg.2017.10.024 29361479 PMC6043421

[35] Cannatà A, De Angelis G, Boscutti A, Normand C, Artico J, Gentile P, et al. Arrhythmic risk stratification in non-ischaemic dilated cardiomyopathy beyond ejection fraction. Heart. 2020;106(9):656–64. doi: 10.1136/heartjnl-2019-315942 31964657

[36] Leyva F, Taylor RJ, Foley PWX, Umar F, Mulligan LJ, Patel K, et al. Left ventricular midwall fibrosis as a predictor of mortality and morbidity after cardiac resynchronization therapy in patients with nonischemic cardiomyopathy. J Am Coll Cardiol. 2012;60(17):1659–67. doi: 10.1016/j.jacc.2012.05.054 23021326

[37] Puntmann VO, Carr-White G, Jabbour A, Yu C-Y, Gebker R, Kelle S, et al. T1-Mapping and outcome in nonischemic cardiomyopathy: All-cause mortality and heart failure. JACC Cardiovasc Imaging. 2016;9(1):40–50. doi: 10.1016/j.jcmg.2015.12.001 26762873

[38] Vita T, Gräni C, Abbasi SA, et al. Comparing CMR mapping methods and myocardial patterns toward heart failure outcomes in nonischemic dilated cardiomyopathy. JACC Cardiovasc Imaging. 2019;12(8 Pt 2):1659–69. doi: 10.1016/j.jcmg.2018.08.021 30448130 PMC6506397

[39] Nakamori S, Ngo LH, Rodriguez J, Neisius U, Manning WJ, Nezafat R. T1 mapping tissue heterogeneity provides improved risk stratification for ICDs without needing gadolinium in patients with dilated cardiomyopathy. JACC Cardiovasc Imaging. 2020;13(9):1917–30. doi: 10.1016/j.jcmg.2020.03.014 32653543

[40] Li Y, Xu Y, Li W, Guo J, Wan K, Wang J, et al. Cardiac MRI to predict sudden cardiac death risk in dilated cardiomyopathy. Radiology. 2023;307(3):e222552. doi: 10.1148/radiol.222552 36916890

