# Supplementary material for: Prognostic value of cardiac magnetic resonance–derived global longitudinal strain in LGE-negative dilated cardiomyopathy
Source: PLoS One. 2026 Mar 27;21(3):e0345077. doi: 10.1371/journal.pone.0345077 (PMC13028522; doi:10.1371/journal.pone.0345077)
Supplement: S1 File — (DOCX) [file pone.0345077.s001.docx]

| **S1 Table. Primary endpoint: Incremental Cox models (Age + NYHA class + remodeling indices) and incremental value of GLS** | | | | |
| --- | --- | --- | --- | --- |
| **Age+NYHA+LAD** |  | | | |
|  | Base HR (95% CI) | Base P | +GLS HR (95% CI) | +GLS P |
| Age (per year) | 1.03 (1.01–1.06) | 0.015 | 1.03 (1.01–1.05) | 0.014 |
| NYHA class III/IV vs I/II | 2.35 (1.17–4.72) | 0.016 | 1.74 (0.87–3.50) | 0.117 |
| LAD (mm) | 1.03 (1.00–1.06) | 0.087 | 1.01 (0.98–1.05) | 0.465 |
| GLS (%; less negative = worse) |  |  | 1.43 (1.18–1.74) | <0.001 |
| N (events) | 378 (35) |  | 378 (35) |  |
| Harrell C-index (apparent) | 0.685 |  | 0.759 |  |
| ΔC (+GLS − Base) |  |  | +0.074 |  |
| Likelihood-ratio test (Base vs +GLS) |  |  | χ²=17.209 | <0.001 |
| **Age+NYHA+LVmassi** |  | | | |
|  | Base HR (95% CI) | Base P | +GLS HR (95% CI) | +GLS P |
| Age (per year) | 1.03 (1.01–1.06) | 0.006 | 1.03 (1.01–1.06) | 0.006 |
| NYHA class III/IV vs I/II | 2.00 (0.98–4.08) | 0.056 | 1.69 (0.85–3.37) | 0.132 |
| LV mass index (g/m²) | 1.02 (1.01–1.03) | 0.005 | 1.01 (1.00–1.03) | 0.056 |
| GLS (%; less negative = worse) |  |  | 1.41 (1.17–1.72) | <0.001 |
| N (events) | 378 (35) |  | 378 (35) |  |
| Harrell C-index (apparent) | 0.692 |  | 0.767 |  |
| ΔC (+GLS − Base) |  |  | +0.075 |  |
| Likelihood-ratio test (Base vs +GLS) |  |  | χ²=15.804 | <0.001 |
| **Age+NYHA+LVEDVi** |  | | | |
|  | Base HR (95% CI) | Base P | +GLS HR (95% CI) | +GLS P |
| Age (per year) | 1.03 (1.01–1.06) | 0.006 | 1.04 (1.01–1.06) | 0.003 |
| NYHA class III/IV vs I/II | 1.99 (1.00–3.96) | 0.049 | 1.77 (0.89–3.50) | 0.102 |
| LVEDVi (mL/m²) | 1.01 (1.01–1.01) | <0.001 | 1.01 (1.01–1.01) | <0.001 |
| GLS (%; less negative = worse) |  |  | 1.35 (1.11–1.63) | 0.002 |
| N (events) | 378 (35) |  | 378 (35) |  |
| Harrell C-index (apparent) | 0.770 |  | 0.793 |  |
| ΔC (+GLS − Base) |  |  | +0.023 |  |
| Likelihood-ratio test (Base vs +GLS) |  |  | χ²=11.394 | <0.001 |
| Abbreviations as Tables 1. | | | | |

| **S2 Table. Primary endpoint: Incremental Cox models (log NT-proBNP + remodeling indices) and incremental value of GLS** | | | | |
| --- | --- | --- | --- | --- |
| **logBNP+LAD** |  | | | |
|  | Base HR (95% CI) | Base P | +GLS HR (95% CI) | +GLS P |
| log NT-proBNP | 2.17 (1.21–3.91) | 0.010 | 1.73 (0.96–3.11) | 0.066 |
| LAD (mm) | 1.03 (1.00–1.07) | 0.038 | 1.02 (0.99–1.05) | 0.244 |
| GLS (%; less negative = worse) |  |  | 1.41 (1.16–1.71) | <0.001 |
| N (events) | 378 (35) |  | 378 (35) |  |
| Harrell C-index (apparent) | 0.640 |  | 0.735 |  |
| ΔC (+GLS − Base) |  |  | +0.095 |  |
| Likelihood-ratio test (Base vs +GLS) |  |  | χ²=16.714 | <0.001 |
| **logBNP+LVmassi** |  | | | |
|  | Base HR (95% CI) | Base P | +GLS HR (95% CI) | +GLS P |
| log NT-proBNP | 2.14 (1.18–3.88) | 0.013 | 1.77 (0.98–3.21) | 0.058 |
| LV mass index (g/m²) | 1.02 (1.01–1.03) | 0.003 | 1.01 (1.00–1.03) | 0.080 |
| GLS (%; less negative = worse) |  |  | 1.39 (1.14–1.68) | <0.001 |
| N (events) | 378 (35) |  | 378 (35) |  |
| Harrell C-index (apparent) | 0.625 |  | 0.736 |  |
| ΔC (+GLS − Base) |  |  | +0.111 |  |
| Likelihood-ratio test (Base vs +GLS) |  |  | χ²=14.561 | <0.001 |
| **logBNP+LVEDVi** |  | | | |
|  | Base HR (95% CI) | Base P | +GLS HR (95% CI) | +GLS P |
| log NT-proBNP | 1.62 (0.91–2.88) | 0.098 | 1.48 (0.84–2.60) | 0.174 |
| LVEDVi (mL/m²) | 1.01 (1.01–1.01) | <0.001 | 1.01 (1.00–1.01) | <0.001 |
| GLS (%; less negative = worse) |  |  | 1.33 (1.10–1.60) | 0.003 |
| N (events) | 378 (35) |  | 378 (35) |  |
| Harrell C-index (apparent) | 0.748 |  | 0.774 |  |
| ΔC (+GLS − Base) |  |  | +0.026 |  |
| Likelihood-ratio test (Base vs +GLS) |  |  | χ²=11.037 | <0.001 |
| Abbreviations as Tables 1. | | | | |

| **S3 Table. Secondary endpoint: Incremental Cox models (Age + NYHA class + remodeling indices) and incremental value of GLS** | | | | |
| --- | --- | --- | --- | --- |
| **Age+NYHA+LAD** |  | | | |
|  | Base HR (95% CI) | Base P | +GLS HR (95% CI) | +GLS P |
| Age (per year) | 1.02 (1.00–1.04) | 0.022 | 1.02 (1.00–1.04) | 0.020 |
| NYHA class III/IV vs I/II | 2.14 (1.32–3.47) | 0.002 | 1.75 (1.07–2.86) | 0.026 |
| LAD (mm) | 1.02 (1.00–1.05) | 0.019 | 1.02 (1.00–1.04) | 0.099 |
| GLS (%; less negative = worse) |  |  | 1.19 (1.07–1.33) | 0.002 |
| N (events) | 378 (72) |  | 378 (72) |  |
| Harrell C-index (apparent) | 0.662 |  | 0.700 |  |
| ΔC (+GLS − Base) |  |  | +0.038 |  |
| Likelihood-ratio test (Base vs +GLS) |  |  | χ²=11.134 | <0.001 |
| **Age+NYHA+LVmassi** |  | | | |
|  | Base HR (95% CI) | Base P | +GLS HR (95% CI) | +GLS P |
| Age (per year) | 1.02 (1.01–1.04) | 0.010 | 1.02 (1.01–1.04) | 0.010 |
| NYHA class III/IV vs I/II | 2.01 (1.23–3.28) | 0.005 | 1.74 (1.07–2.83) | 0.025 |
| LV mass index (g/m²) | 1.01 (1.00–1.02) | 0.008 | 1.01 (1.00–1.02) | 0.067 |
| GLS (%; less negative = worse) |  |  | 1.19 (1.06–1.33) | 0.002 |
| N (events) | 378 (72) |  | 378 (72) |  |
| Harrell C-index (apparent) | 0.663 |  | 0.701 |  |
| ΔC (+GLS − Base) |  |  | +0.038 |  |
| Likelihood-ratio test (Base vs +GLS) |  |  | χ²=10.425 | 0.001 |
| **Age+NYHA+LVEDVi** |  | | | |
|  | Base HR (95% CI) | Base P | +GLS HR (95% CI) | +GLS P |
| Age (per year) | 1.02 (1.01–1.04) | 0.007 | 1.02 (1.01–1.04) | 0.005 |
| NYHA class III/IV vs I/II | 1.98 (1.23–3.19) | 0.005 | 1.73 (1.07–2.80) | 0.024 |
| LVEDVi (mL/m²) | 1.01 (1.00–1.01) | <0.001 | 1.01 (1.00–1.01) | <0.001 |
| GLS (%; less negative = worse) |  |  | 1.16 (1.03–1.29) | 0.011 |
| N (events) | 378 (72) |  | 378 (72) |  |
| Harrell C-index (apparent) | 0.685 |  | 0.704 |  |
| ΔC (+GLS − Base) |  |  | +0.018 |  |
| Likelihood-ratio test (Base vs +GLS) |  |  | χ²=7.243 | 0.007 |
| Abbreviations as Tables 1. | | | | |

| **S4 Table. Secondary endpoint: Incremental Cox models (log NT-proBNP + remodeling indices) and incremental value of GLS** | | | | |
| --- | --- | --- | --- | --- |
| **logBNP+LAD** |  | | | |
|  | Base HR (95% CI) | Base P | +GLS HR (95% CI) | +GLS P |
| log NT-proBNP | 2.52 (1.65–3.84) | <0.001 | 2.21 (1.45–3.38) | <0.001 |
| LAD (mm) | 1.03 (1.00–1.05) | 0.016 | 1.02 (1.00–1.04) | 0.077 |
| GLS (%; less negative = worse) |  |  | 1.17 (1.05–1.30) | 0.004 |
| N (events) | 378 (72) |  | 378 (72) |  |
| Harrell C-index (apparent) | 0.668 |  | 0.701 |  |
| ΔC (+GLS − Base) |  |  | +0.033 |  |
| Likelihood-ratio test (Base vs +GLS) |  |  | χ²=9.628 | 0.002 |
| **logBNP+LVmassi** |  | | | |
|  | Base HR (95% CI) | Base P | +GLS HR (95% CI) | +GLS P |
| log NT-proBNP | 2.55 (1.66–3.91) | <0.001 | 2.29 (1.50–3.50) | <0.001 |
| LV mass index (g/m²) | 1.01 (1.00–1.02) | 0.007 | 1.01 (1.00–1.02) | 0.067 |
| GLS (%; less negative = worse) |  |  | 1.16 (1.04–1.30) | 0.006 |
| N (events) | 378 (72) |  | 378 (72) |  |
| Harrell C-index (apparent) | 0.673 |  | 0.703 |  |
| ΔC (+GLS − Base) |  |  | +0.030 |  |
| Likelihood-ratio test (Base vs +GLS) |  |  | χ²=8.696 | 0.003 |
| **logBNP+LVEDVi** |  | | | |
|  | Base HR (95% CI) | Base P | +GLS HR (95% CI) | +GLS P |
| log NT-proBNP | 2.23 (1.46–3.40) | <0.001 | 2.05 (1.35–3.13) | <0.001 |
| LVEDVi (mL/m²) | 1.01 (1.00–1.01) | <0.001 | 1.00 (1.00–1.01) | <0.001 |
| GLS (%; less negative = worse) |  |  | 1.15 (1.03–1.28) | 0.012 |
| N (events) | 378 (72) |  | 378 (72) |  |
| Harrell C-index (apparent) | 0.699 |  | 0.714 |  |
| ΔC (+GLS − Base) |  |  | +0.014 |  |
| Likelihood-ratio test (Base vs +GLS) |  |  | χ²=7.148 | 0.008 |
| Abbreviations as Tables 1. | | | | |

| **S5 Table. Apparent and optimism-corrected C-indices with bootstrap internal validation for all developed models** | | | |
| --- | --- | --- | --- |
| **Model** | **Description** | **Apparent C-index (95% CI)** | **Optimism-corrected C-index (95% CI)** |
| **Model 1** | GLS | 0.681 (0.632–0.730) | 0.681 (0.636–0.734) |
| **Model 2** | LVEF | 0.714 (0.634–0.794) | 0.711 (0.640–0.791) |
| **Model 3** | GLS + LVEF | 0.756 (0.689–0.822) | **0.754 (0.689–0.822)** |
| **Model 4** | GLS + LVEF + Age + NYHA | 0.801 (0.729–0.873) | **0.785 (0.719–0.858)** |
| Abbreviations as Tables 1. | | | |

| **S6 Table. Pairwise Bootstrap Comparison of Model Performance Differences** | | | | | |
| --- | --- | --- | --- | --- | --- |
| **Contrast** | **Metric** | **mean ΔC** | **95% CI** | **p-value** | **Holm-adjusted p** |
| **Model 1 vs. Model 2** | Apparent C-index | 0.041 | 0.002 – 0.097 | 0.310 | 0.310 |
|  | Optimism-corrected C-index | **0.031** | **0.001** – **0.099** | **0.386** | **0.488** |
| **Model 1 vs. Model 3** | Apparent C-index | 0.076 | 0.028 – 0.120 | < 0.001 | < 0.001 |
|  | Optimism-corrected C-index | **0.073** | **0.030** – **0.122** | **0.014** | **0.048** |
| **Model 2 vs. Model 3** | Apparent C-index | 0.041 | 0.016 – 0.066 | < 0.001 | < 0.001 |
|  | Optimism-corrected C-index | **0.042** | **0.017** – **0.067** | **0.012** | **0.048** |
| **Model 3 vs. Model 4** | Apparent C-index | 0.051 | 0.009 – 0.104 | 0.010 | 0.020 |
|  | Optimism-corrected C-index | **0.036** | **0.002** – **0.076** | **0.244** | **0.488** |
| **In comparisons labeled “Model X vs Model Y,” Model X (on the left) represents the older model, while Model Y (on the right) represents the newer model. Model 1= GLS; 2= LVEF; Model 3= GLS + LVEF; Model 4= GLS + LVEF. Abbreviations as Tables 1.** | | | | | |
